# Supplementary material for: Neuron-specific chromatin disruption at CpG islands and aging-related regions in Kabuki syndrome mice
Source: bioRxiv. 2023 Aug 3:2023.08.01.551456. Preprint. [Version 1] doi: 10.1101/2023.08.01.551456 (PMC10418197; doi:10.1101/2023.08.01.551456)
Supplement: Supplement 15 [file NIHPP2023.08.01.551456v1-supplement-15.pdf]

bioRxiv preprint doi: https://doi.org/10.1101/2023.08.01.551456; this version posted August 3, 2023. The copyright holder for this preprint (which was not certified by peer review) is the author/funder, who has granted bioRxiv a license to display the preprint in perpetuity. It is made available under aCC-BY 4.0 International license.

SUPPLEMENTARY MATERIALS

Neuron-specific chromatin disruption at CpG islands and aging-related regions in Kabuki syndrome mice

Leandros Boukas*, Teresa Romeo Luperchio*, Afrooz Razi, Kasper D. Hansen**, Hans T. Bjornsson**

* Shared first-author contribution
** Correspondence to khansen@jhsph.edu (KDH), hbjorns1@jhmi.edu (HTB)

Contents

1 Supplemental Figures **2**

2 List of Supplementary Tables **9**

bioRxiv preprint doi: https://doi.org/10.1101/2023.08.01.551456; this version posted August 3, 2023. The copyright holder for this preprint (which was not certified by peer review) is the author/funder, who has granted bioRxiv a license to display the preprint in perpetuity. It is made available under aCC-BY 4.0 International license.

1 Supplemental Figures

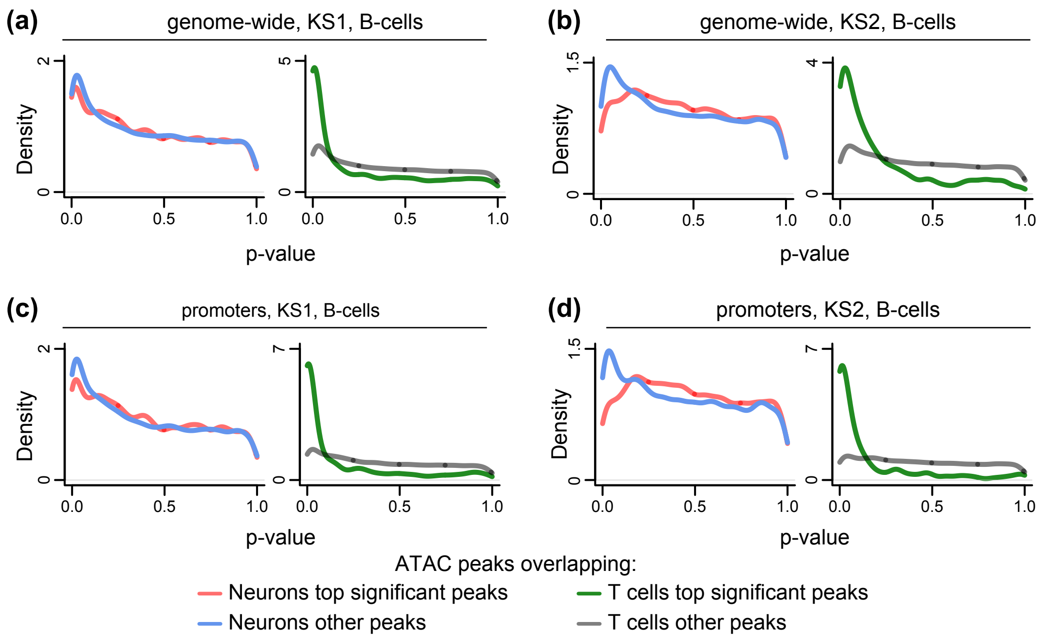

Supplementary Figure S1. Evaluating whether neurons, B cells and T cells exhibit the same changes in chromatin accessibility in Kabuki syndrome types 1 and 2; alternate conditioning. **(a) -(d)** Like Figure 1a, but where the x-axis corresponds to p-values from the mutant (KS1 or KS2) vs wild-type differential analysis in B cells, and the conditioning (i.e. the stratification into red vs blue or green vs gray densities) is done either based on which peaks overlap peaks with disrupted accessibility in neurons (red vs blue), or based on which peaks overlap peaks with disrupted accessibility in T cells (green vs gray).

bioRxiv preprint doi: https://doi.org/10.1101/2023.08.01.551456; this version posted August 3, 2023. The copyright holder for this preprint (which was not certified by peer review) is the author/funder, who has granted bioRxiv a license to display the preprint in perpetuity. It is made available under aCC-BY 4.0 International license.

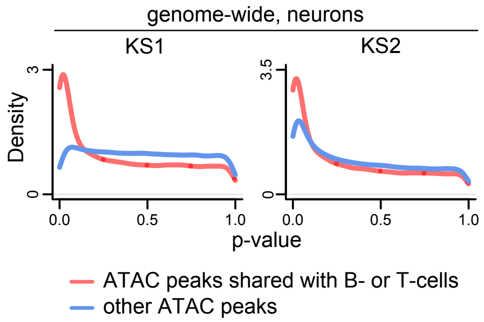

Supplementary Figure S2. Evaluating the relationship between probability of differential accessibility in KS1 and KS2 neurons and neuron-specific vs broad peak activity. The distributions of p-values from the KS1 and KS2 mutant vs wild-type differential accessibility analyses in neurons, stratified according to whether the ATAC peaks are specific to neurons (blue densities) or overlap peaks that are also present in B or T cells (red densities).

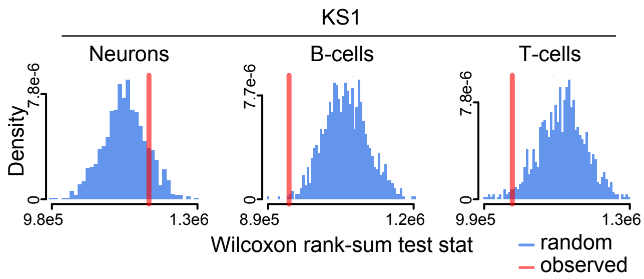

Supplementary Figure S3. Investigating the chromatin disruption of promoters of mouse orthologs of human episignature genes. The observed value (red vertical line) of the Wilcoxon rank-sum test statistic obtained after comparing the p-values corresponding to mouse orthologs of human genes whose promoters contain CpGs differentially methylated between KS1 patients and controls in whole blood to p-values corresponding to all other genes. See Methods for details. Observed values lower than expected under the null (blue distributions) indicate the collective disruption of aging-related peaks. The observed value (red vertical line) was obtained by comparing The null distributions were obtained by repeated random sampling from all genes to obtain a null gene set of equal size to the set of episignature homolog genes and compare it to all other genes.

bioRxiv preprint doi: https://doi.org/10.1101/2023.08.01.551456; this version posted August 3, 2023. The copyright holder for this preprint (which was not certified by peer review) is the author/funder, who has granted bioRxiv a license to display the preprint in perpetuity. It is made available under aCC-BY 4.0 International license.

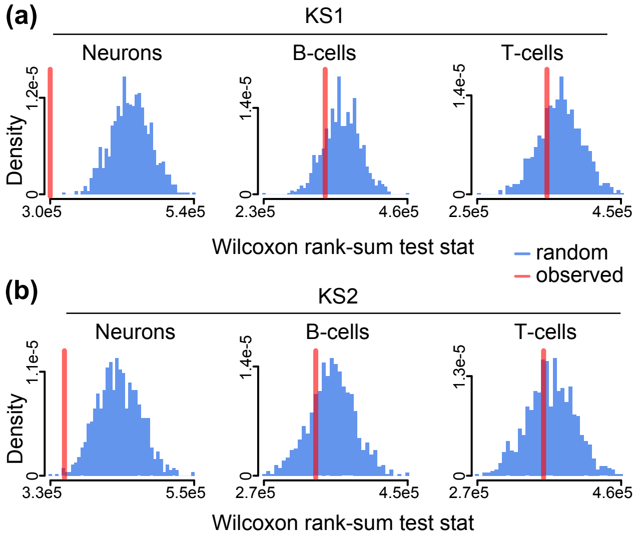

Supplementary Figure S4. Investigating the chromatin disruption of promoters of longevity-associated genes in neurons, B cells and T cells in Kabuki syndrome types 1 and 2. (a)-(b) Like Figure 4a, but for ATAC peaks overlapping promoters of genes in the mouse KEGG "longevity regulating pathway". Shown for KS1 and KS2. See Methods for details.

bioRxiv preprint doi: https://doi.org/10.1101/2023.08.01.551456; this version posted August 3, 2023. The copyright holder for this preprint (which was not certified by peer review) is the author/funder, who has granted bioRxiv a license to display the preprint in perpetuity. It is made available under aCC-BY 4.0 International license.

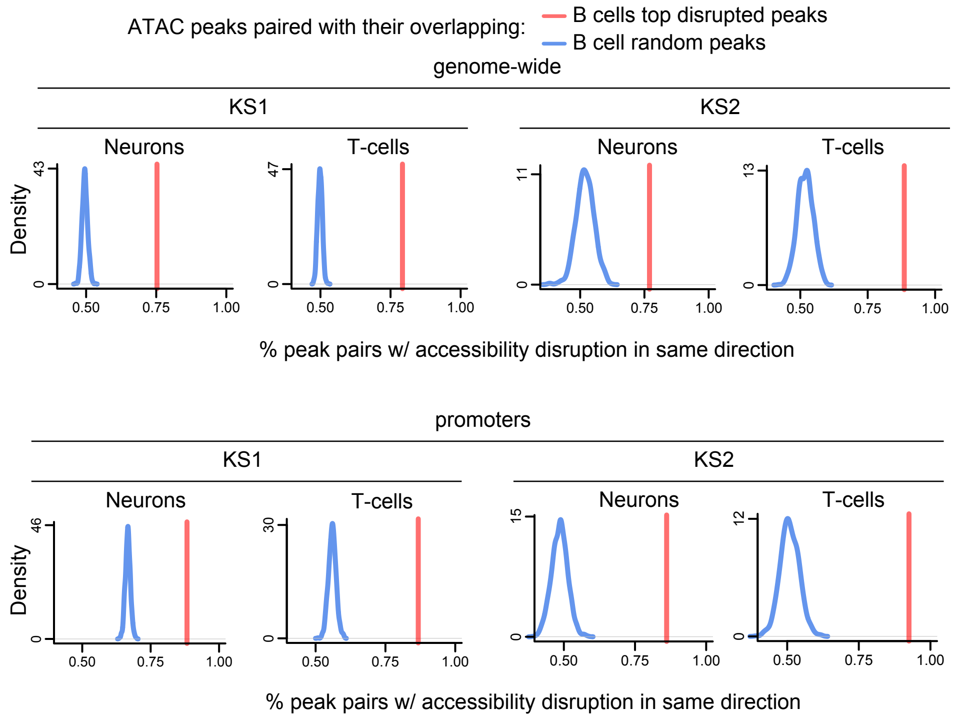

Supplementary Figure S5. Evaluating whether chromatin accessibility changes are towards the same direction in neurons, B cells and T cells in Kabuki syndrome types 1 and 2; null distributions vs observed values. The red vertical lines depict the observed percentage of ATAC peaks (either in neurons or T cells) that overlap the top significantly disrupted peaks in B cells (qvalue < 0.1) and show accessibility changes towards the same direction. The blue densities correspond to null distributions, obtained by repeatedly (1000 times) sampling a random set of B cell peaks and computing the percentage of overlapping neuronal peaks that show accessibility changes towards the same direction. The sampling is performed so as to ensure that, among the random peaks, the balance of peaks with increased vs decreased accessibility in mutants vs wild-type is the same as among the top disrupted B cell peaks.

bioRxiv preprint doi: https://doi.org/10.1101/2023.08.01.551456; this version posted August 3, 2023. The copyright holder for this preprint (which was not certified by peer review) is the author/funder, who has granted bioRxiv a license to display the preprint in perpetuity. It is made available under aCC-BY 4.0 International license.

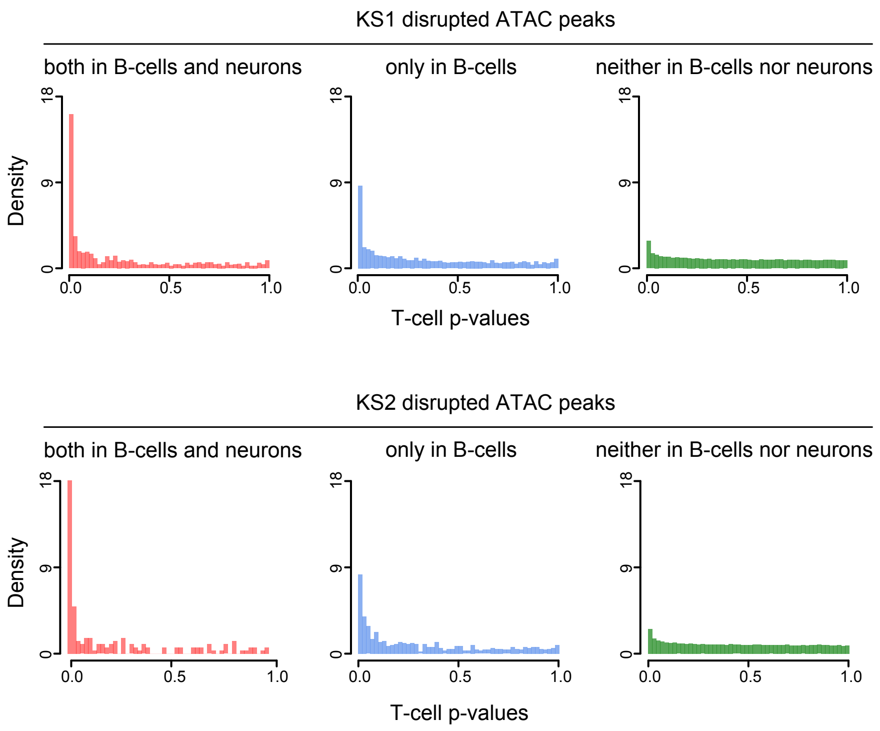

Supplementary Figure S6. Evaluating whether neuronal chromatin disruption can distinguish peaks disrupted in T cells on top of information provided by disruption in B cells. The p-value distributions of T cell ATAC peaks that overlap peaks that are: differential in both B and neurons; differential in B cells but not in neurons; neither differential in B cells nor neurons. Depicted separately for KS1 and KS2.

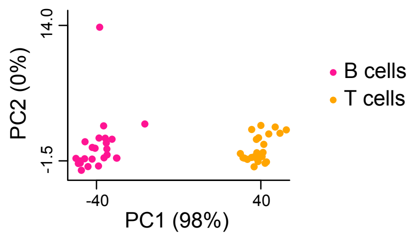

Supplementary Figure S7. Joint principal component analysis of samples from B and T cells of KS1, KS2, and WT mice. Each point corresponds to a single sample, and points are colored according to whether they come from B or T cells. All 3 mouse genotypes (KS1, KS2, wild-type) are represented among these samples. The x and y axes correspond to the first and second, respectively, principal component; the percentage of variance each explains is indicated in the parentheses. Note that since almost all of the variance is explained by PC1, between-sample distances along the y axis are much smaller than distances along the x axis, even when they visually appear similar.

bioRxiv preprint doi: https://doi.org/10.1101/2023.08.01.551456; this version posted August 3, 2023. The copyright holder for this preprint (which was not certified by peer review) is the author/funder, who has granted bioRxiv a license to display the preprint in perpetuity. It is made available under aCC-BY 4.0 International license.

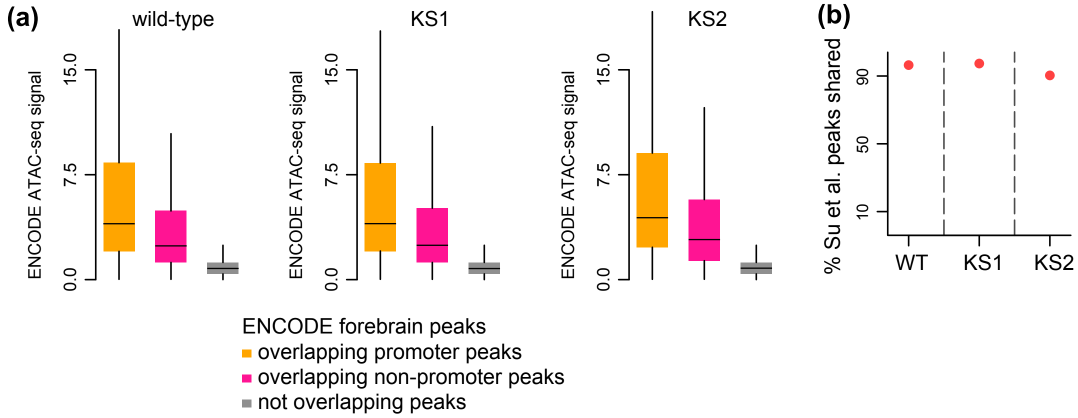

Supplementary Figure S8. Orthogonal validation of ATAC peaks detected in KS1 and KS2 in hippocampal neurons. (a) The distribution of the ATAC signal (enrichment over random regions) of ENCODE peaks detected in post-natal forebrain (Gorkin et al. [53]; see also Methods), stratified according to whether they overlap peaks detected in our study within promoters, overlap peaks detected in our study outside promoters, or do not overlap peaks detected in our study. Shown for wild-type, KS1, and KS2. **(b)** The y axis depicts the percentage of ATAC peaks detected in Su et al. [66] that are also detected in neurons in our study (see also Methods), separately for each of the 3 genotypes.

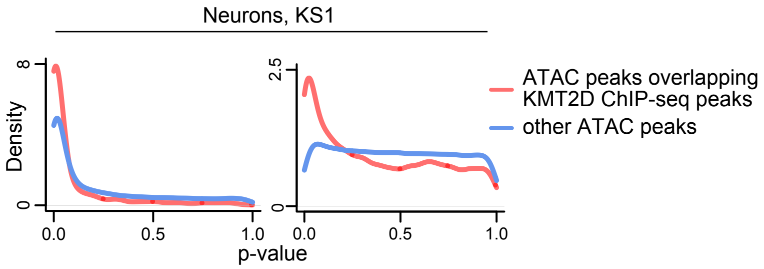

Supplementary Figure S9. Evaluating the overlap between regions that exhibit changes in neuronal chromatin accessibility in Kabuki syndrome types 1 and regions bound by KMT2D in mouse ht22 cells. The distributions of p-values from the KS1 mutant vs wild-type differential accessibility analysis, for neuronal ATAC peaks that do and do not overlap ChIP-seq KMT2D peaks in mouse ht22 cells (a mouse hippocampal neuronal cell line). ATAC peaks in promoters and ATAC peaks outside promoters are depicted separately.

bioRxiv preprint doi: https://doi.org/10.1101/2023.08.01.551456; this version posted August 3, 2023. The copyright holder for this preprint (which was not certified by peer review) is the author/funder, who has granted bioRxiv a license to display the preprint in perpetuity. It is made available under aCC-BY 4.0 International license.

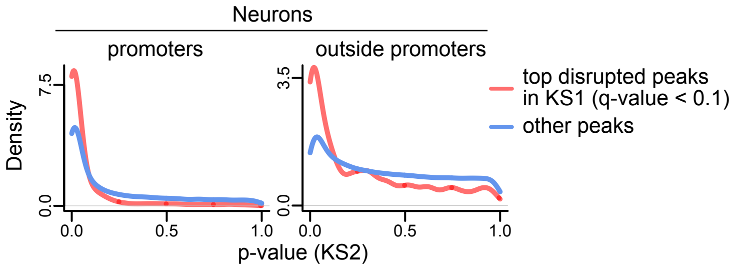

Supplementary Figure S10. Evaluating the overlap between the regions with differential accessibility in KS1 and KS2 neurons. The distributions of p-values from the KS2 mutant vs wild-type differential accessibility analysis, stratified according to whether the same neuronal peaks are disrupted in KS1 neurons (red densities; q-value ¡ 0.1 in KS1) or not (blue densities; rest of the p-values). Shown separately for peaks inside promoters and peaks outside promoters.

bioRxiv preprint doi: https://doi.org/10.1101/2023.08.01.551456; this version posted August 3, 2023. The copyright holder for this preprint (which was not certified by peer review) is the author/funder, who has granted bioRxiv a license to display the preprint in perpetuity. It is made available under aCC-BY 4.0 International license.

2 List of Supplementary Tables

1. **Supplemental Table 1**: The coordinates (in mm10), log-fold changes, p-values, and q-values of the most significantly disrupted ATAC peaks (qvalue ≤ 0.1) from the KS1 vs wild-type differential accessibility analysis in neurons.
2. **Supplemental Table 2**: Like Supplemental Table 1, but for KS2.
3. **Supplemental Table 3**: The coordinates (in mm10), log-fold changes, p-values, and q-values of ATAC peaks overlapping promoters of epigenetic machinery genes from the KS1 vs wild-type differential accessibility analysis in neurons.
4. **Supplemental Table 4**: Like Supplemental Table 3, but for KS2.
5. **Supplemental Table 5**: The top 20 significant Reactome pathways based on the disruption of promoter ATAC peaks in the KS1 vs wild-type differential accessibility analysis in neurons.
6. **Supplemental Table 6**: Like Supplemental Table 5, but for KS2.
7. **Supplemental Table 7**: The gene names and ENSEMBL id's of genes downstream of promoter ATAC peaks that exhibit disrupted accessibility in neurons, B and T cells at the 10% FDR level in KS1. If more than 1 gene are downstream of the same promoter (e.g. in cases of bidirectional promoters), all these genes are separately provided.
8. **Supplemental Table 8**: The coordinates (in mm10), log-fold changes, p-values, and q-values from the KS1 vs wild-type differential accessibility analysis in B cells of ATAC peaks that exhibit disrupted accessibility in all three cell types.
9. **Supplemental Table 9**: Like Supplemental Table 8, but from the KS1 vs wild-type differential accessibility analysis in T cells.
10. **Supplemental Table 10**: Like Supplemental Table 8, but from the KS1 vs wild-type differential accessibility analysis in neurons.
11. **Supplemental Table 11**: Like Supplemental Table 7, but for KS2.
12. **Supplemental Table 12**: Like Supplemental Table 8, but from the KS2 vs wild-type differential accessibility analysis.
13. **Supplemental Table 13**: Like Supplemental Table 9, but from the KS2 vs wild-type differential accessibility analysis.
14. **Supplemental Table 14**: Like Supplemental Table 10, but from the KS2 vs wild-type differential accessibility analysis.
